# Predictor variables of abnormal imaging findings of syncope in the emergency department

**DOI:** 10.1186/s12245-018-0180-0

**Published:** 2018-03-12

**Authors:** Kerem Ozturk, Esra Soylu, Cem Bilgin, Bahattin Hakyemez, Mufit Parlak

**Affiliations:** 1 Department of Radiology, Faculty of Medicine, Uludag University, Bursa, Turkey; 2 Radiology Clinic, Cekirge State Hospital, Bursa, Turkey

**Keywords:** Computed tomography (CT), Magnetic resonance imaging (MRI), Syncope, Emergency department (ED)

## Abstract

**Background:**

This study aimed to describe the pathological findings and to analyze clinical predictors of abnormal imaging findings in patients presenting to the emergency department (ED) with syncope.

**Methods:**

The database was retrospectively reviewed for all patients who underwent cranial computed tomography (CT) or magnetic resonance imaging (MRI), having the symptom of syncope. Patients were included only if they were from the emergency department and excluded if were under 18 years of age, had known recent intracranial pathology, known brain tumor, or having a history of trauma. The primary outcome was assumed as abnormal head CT or MRI including intracranial hemorrhage, acute or subacute stroke, and newly diagnosed brain mass. Univariate and multivariate logistic regression analysis was utilized to determine the association between clinical variables and any significant pathology in either CT or MR scan.

**Results:**

Total of 1230 syncope (717 men and 513 women; range, 18–92 years; mean, 54.5 years) as presenting symptoms were identified in patients receiving either cranial CT or MR scan in the ED. Abnormal findings related to the syncope were observed in 47 (3.8%) patients. The following predictor variables were found to be significantly correlated with acutely abnormal head CT and MRI: a focal neurologic deficit, history of malignancy, hypertension, and age greater than 60 years.

**Conclusions:**

Our data offer that the identification of predictor variables has a potential to decrease the routine use of head CT and MRI in patients admitting to the ED with syncope.

## Background

Syncope, defined as a transient loss of consciousness, is a common clinical condition accounting for up to 3% of all emergency department (ED) visits and 6% of hospital admissions [1–4]. The identification of life-threatening conditions is challenging for the emergency physician [5]. In spite of detailed investigation, the specific cause could not be determined approximately 40% of cases [6, 7].

Along with these concerns, a reevaluation of the practice to obtain routine imaging methods in patients with syncope is required to reduce unnecessary and expensive medical testing [8]. Although computed tomographic (CT) and magnetic resonance imaging (MRI) scans of the head are often routinely utilized, only a few studies have examined their value to determine the severity of the disorders [9, 10]. Determining the clinical and laboratory variables associated with these decisions is a step toward progress for the assessment of patients admitting to the ED with syncope [11].

Therefore, we investigated consecutive patients who underwent head CT and MRI in the emergency department for syncope. Our purpose was to describe the pathological findings and to analyze predictor variables of pathological imaging findings in patients presenting to the emergency department with syncope.

## Methods

### Study design and setting

This retrospective study was approved by our hospital’s institutional review board with a waiver of informed consent (Committee reference number 2016-16/11) and was conducted in compliance with Health Insurance Portability and Accountability Act guidelines.

This study was conducted in a university hospital with 150,000 adult patient emergency visits annually**.** Our institution’s picture archiving and communication system (Centricity; GE Healthcare, Little Chalfont, UK) was used to conduct a search for patients having a cranial CT or MRI between 1 January 2011 and 1 December 2016. The database was investigated for all patients who visited the ED with a symptom containing any of the following terms: syncope, fainting, passing out, and loss of consciousness. The patient history and clinical information were gathered from electronic medical record system. The risk factors were evaluated by the in-depth evaluation of the initial electronic patient notes recorded by the ED physician or physician assistant including vital signs and laboratory findings.

Clinical variables that were missing from or not referred in the electronic patient note were considered as not present. Patients with multiple head CT and MRI during the study period were included only once.

Patients were excluded from the study if primary symptoms did not consist of any of the designated search terms, were under 18 years of age, were not from the ED, presented due to a history of trauma, had existing intracranial pathology, and had a known primary or metastatic brain tumor. Persistent altered mental status, alcohol, or illicit drug-related loss of consciousness, seizure, and coma were also excluded. Patients with a history of malignancy without metastatic tumor in the brain were still included into the study. Visits to other hospitals during time period were also not included to the study. Electrocardiogram (ECG) findings were gathered from patient reports retrospectively. Electrocardiography findings were available in only 168 out of 1230 (13%) syncope patients; a concerning finding including ST-segment changes, atrial fibrillation/flutter, or second- or third-degree heart block, was reported in 13 (8%) patients. The ECG data were not sufficient to be used as a factor in statistical analyses.

In our department, cranial MRI is utilized following head CT imaging if considered necessary in patients admitted to the ED with syncope. Patients with an initially negative CT and subsequent positive MRI are included as a positive MRI finding. Whereas, if pathology is identified on head CT scan initially, consequent cranial MRI is excluded from the study to obviate sample bias.

### Outcome measures

Cranial CT and MRI studies were interpreted by board-certified neuro-radiologists with 15 and 25 years’ experience, consecutively. The referring emergency physician did not play a role in image interpretation. The primary outcomes were abnormal cranial CT or MRI findings characterized by an acute intracranial hemorrhage, acute or subacute infarct, a newly diagnosed brain mass or other clinically important abnormality that required intervention. According to the results gathered from previous studies [10, 12], the following risk factors were identified: sex, age, focal neurologic deficit, history of malignancy, history of drug abuse including alcohol, fever or leukocytosis, hypertension, diabetes mellitus, disturbances in coagulation profile, nausea or vomiting, and laboratory findings of metabolic derangement such as hypoglycemia. Cranial CT and MRI examinations were considered abnormal only if patient’s condition was related to the imaging finding.

Coagulopathy included abnormal international normalized ratio (INR), abnormal partial thromboplastin time (PTT), abnormal prothrombin time (PT), thrombocytopenia, active anticoagulant therapy, and history of the coagulopathic disorder (e.g., hemophilia, Factor V Leiden).

Vital signs were considered to be normal if the patient’s temperature was between 35 °C (95 °F) and 38 °C (100.4 °F), pulse 60–100 beats/min, respiration 14 to 20 breaths/min, blood pressure ranging from systolic blood pressure 90 to 140 mmHg, oxygen saturation over 90% room air.

Syncope was defined as a transient loss of consciousness with a brief period of unresponsiveness and a loss of postural tone, resulting in spontaneous recovery without any resuscitation.

### Statistical analysis

All statistical comparisons were performed by using the software of Statistical Package for the Social Sciences (SPSS, version 23.0; SPSS, Chicago, IL). We examined separately the distribution of associated symptoms (predictive variables) in the different categories. The discrepancy in their distribution was analyzed with the *x*^2^ test and, when expected frequencies were < 5 for any categories, the Fisher’s exact test was used. Univariate logistic regression analysis was utilized to identify the predictor variables of abnormal cranial CT and MRI findings. Those that were significantly associated with the primary outcome in univariate analysis were also processed with multivariate regression analysis. The strength of association of each predictor variable with the primary outcome was expressed as an odds ratio and 95% confidence interval. A *p* value of less than 0.05 was considered as the statistically significant difference.

## Results

Total of 24,210 head CT and 4502 cranial MRI were searched in this study, and total of 1230 (717 men and 513 women; range, 18–92 years; mean, 54.5 years) syncope as presenting symptoms were identified in patients received either cranial CT or MR scan in the ED from the radiology database. Abnormal findings related to the syncope were observed in 47 (3.8%) (95% CI = 2.5–4.7%) patients.

Twenty-nine patients (2.7%) (95% CI = 1.8–3.6%) had a pathological imaging findings on cranial CT in the 1060 patients who presented to the ED with syncope (Table 1). Using the data from all 1060 patients, univariate and multivariate logistic regression was performed with all determined 11 independent predictor variables (Table 2). Intracranial pathologies on CT were found to be related to the focal neurologic deficit, history of malignancy, hypertension, and age greater than 60 years (Table 3).

**Table 1:** Prevalence of pathological findings in total of 47 patients presenting to the emergency department with syncope

Pathological findings	On CT	On MRI	Total
Infarct	*n* = 8	*n* = 15	*n* = 23
Hemorrhage	*n* = 16	*n* = 2	*n* = 18
Tumor	*n* = 4	*n* = 0	*n* = 4
Subdural effusion	*n* = 1	*n* = 0	*n* = 1
Schizencephaly	*n* = 0	*n* = 1	*n* = 1

*n* number, *CT* computed tomography, *MRI* magnetic resonance imaging

**Table 2:** Frequency of determined predictor variables in 1230 patients with syncope

Predictor variables	Number of patients (%)
Focal neurological deficit	212 (17%)
History of malignancy	122 (10%)
History of drug abuse including alcohol	82 (6%)
Fever or leukocytosis	156 (13%)
Hypertension	138 (11%)
Diabetes mellitus	179 (15%)
Disturbances in coagulation profile	95 (8%)
Nausea or vomiting	191 (16%)
Metabolic derangement	67 (5%)

**Table 3:** Statistically significant predictor variables of pathological findings in 1230 patients presenting to the emergency department with syncope

	Univariate analysis		Multivariate analysis	
	OR (95% Cl)	*p* value	OR (95% Cl)	*p* value
Syncope on CT				
Focal neurological deficit	5.9 (2.8–9.8)	*p* < .001	5.2 (2.3–8.1)	*p* < .001
History of malignancy	5.1 (2.1–8.5)	*p* < .001	4.5 (1.8–6.9)	*p* = 0.005
Hypertension	3.0 (1.3–7.1)	*p* = 0.009	2.1 (0.9–5.7)	*p* = 0.019
Age greater than 60 years	2.6 (1.2–6.7)	*p* = 0.016	1.6 (0.8–5.1)	*p* = 0.036
Syncope on MRI				
Focal neurological deficit	6.8 (4.1–11.3)	*p* < .001	5.7 (3.3–9.4)	*p* < .001
History of malignancy	5.5 (2.3–9.7)	*p* < .001	4.3 (1.7–7.9)	*p* < .001
Age greater than 60 years	2.4 (1.1–8.1)	*p* = 0.018	1.4 (1.0–6.1)	*p* = 0.041

*Cl* confidence interval, *OR* odds ratio, *MRI* magnetic resonance imaging

If patients were imaged only with one or more of the four predictor variables determined by multivariate logistic regression analysis, a sensitivity of 93.1% (95% CI = 88–96.2%) (27 of 29 scans with positive findings) could be obtained, while reducing the number of examinations to 42.9% (95% CI = 37.1–46.3%) of the original number (455 of 1060).

Two patients having syncope would be missed to be selected for CT with using these predictor variables. A 58-year-old man presented to the ED with syncope was found to have a primary glial tumor in the left frontal lobe. He had not any additional neurological symptoms other than syncope. The second patient was a 45-year-old woman who also presented with a syncope and was found to have a small subacute brainstem infarct. Follow-up conventional angiography demonstrated that the patient had vasculitis.

Eighteen patients (10.5%) (95% CI = 6.3–16.8%) were found to have a pathological imaging findings on cranial MRI in the 170 patients who presented to the ED with syncope (Table 1). Using the data from all 170 patients, univariate and multivariate logistic regression was performed with all 11 independent predictor variables (Table 2). Intracranial pathologies on MRI were found to be related to the focal neurologic deficit, history of malignancy, and age greater than 60 years (Table 3).

If patients were imaged only with one or more of these three predictor variables determined by multivariate logistic regression analysis, a sensitivity of 94.4% (95% CI = 85.3–97.8%) (17 of 18 scans with positive findings) could be obtained, while reducing the number of examinations to 60% (95% CI = 53–68.4%) of the original number (102 of 170).

One patient with syncope could be missed to be selected for MRI with using predictor variables. A 38-year-old man with syncope was found to have a schizencephaly without any additional neurological symptoms. However, it is speculated that schizencephaly may be an incidental finding and may not be associated with the clinical symptom of syncope.

## Discussion

The evaluation and management of patients having syncope are demanding for the emergency physician [13, 14]. The physician is confronted with two difficulties in this scenario: first, to quickly identify cases requiring urgent care and secondly, to use resources appropriately. Often, the former becomes the principal goal, and patients receive comprehensive assessment with cross-sectional imaging to exclude serious disorders [15–17].

The more selective use of cross-sectional imaging methods in the ED has a potential to significantly reduce health care costs. However, physicians need reliable guidelines [18, 19] for cranial CT and MRI referral for these patient groups. Screening with cranial CT or MR is utilized in many cases with non-traumatic neurological symptoms, but there are very few studies revealing the efficacy of cranial CT and MR, particularly in the syncope [12, 20]. Based on early data, Kapoor [9] proposed that cranial CT may provide useful information in 4% of patients admitted to the ED with syncope. However, almost all of the abnormal findings in their study were limited to the patients having a focal neurologic deficit or a history of seizure. In a retrospective study, Goyal et al. [7] reported no any pathological head CT findings that were clinically associated with 117 patients presenting to the ED with a syncope. Nevertheless, they excluded patients with competing indications, such as a history of trauma, seizure, mental status changes, and the focal neurologic deficits. Giglio et al. [21] also revealed only one patient with evidence of infarction on head CT out of 44 patients with syncope in the emergency department. Additionally, Grossman et al. [17] reported a diagnostic yield of 5% for the abnormal head CT findings in patients with syncope after excluding the important parameter such as persistent altered mental status, seizure disorder, hypoglycemia, and drug-related or traumatic loss of consciousness. In our study, history of malignancy, focal neurologic deficit, and age greater than 60 years were the most prominent predictor variables of an abnormal cranial CT or MRI. The other variables such as sex, history of drug abuse such as alcohol, fever or leukocytosis, diabetes mellitus, disturbances in coagulation profile, and laboratory findings of metabolic derangement were not found to be independent significant risk factors. However, Wang and You [12] also found altered mental status and derangements in coagulation profile as a factor to be independently predictive. This difference may be partly explained by differences in sample variety in their study group. While our sample size was almost identical with Wang and You, we were also able to focus on specifically non-traumatic neurological symptom of syncope in the ED as well as to define clinical utilization of cranial MRI in patients with syncope.

We evaluated the accuracy and reliability of 11 predictor variables used in the evaluation of patients with syncope and developed a highly sensitive clinical decision rule that may be used to augment physician judgment and allow physicians to rationally decide which patients with syncope may need admission according to their risk for short-term outcomes. Our results correspond with those of other studies [8–10, 17, 20, 21], which found a low diagnostic value for acute intracranial pathologies on head CT and MRI among patients admitted to the emergency department with syncope, despite the more common use of advanced neuroimaging in our population. This finding suggests that common usage of head CT and MRI may not significantly alter the clinical decision of ED physicians. Therefore, we offer that younger patient without focal neurologic deficits or history of malignancy may not benefit from head CT and MRI. The clinical examination and appropriate follow-up may be utilized as an alternative to costly CT or MRI scans for these patients.

Our population differs from those previously studied. The socialized health care system in our country eliminates financial barriers for the most imaging studies. However, we identified only minor differences in the cause of syncope between our study and previous publications [10, 21]. The causes were often of benign in origin and did not lead to the poor outcomes.

Our work adds to this field because it systematically examines and identifies independent predictor variables that are associated with abnormal cranial CT and MRI in patients having syncope. In this study, we could have looked at our data and then derived some predictor variables; however, the purpose of this study was not only to derive a new rule, but also to determine some risk factors based on existing recommendations and evidence. This is a limitation as there are variables in our study that actually may not be predictive or other variables not included that may predict outcome. The strengths of this study include its large sample size, examinations of cranial CT and MRI by neuro-radiologists, detailed investigations of patient risk factors, and associated clinical symptoms.

Our work has some limitations, patient assessment and documentation of clinical findings were not standardized primarily due to retrospective nature, and therefore a prospective validation of the clinical variables are required. In this study, the true effect of utilizing these clinical predictors may not be examined because ED patients without cranial CT and MRI scans were not involved. Although medical records were independently analyzed by neuro-radiologists using all available data, missed diagnoses or delayed complications at other institutions may not have been captured. Additionally, there was no standardized protocol in the ED for the evaluation of patients with syncope. Misclassification of final diagnoses and the potential for underestimating the frequency of delayed presentations of serious neurologic disease is possible. The study was utilized at a single tertiary care academic medical center, and therefore, the results may not be implemented in community hospitals. However, because of our department’s criteria to gather head imaging to the all patients in syncope, it may be supposed that the mix of patients in this study may reflect the population with syncope admitted to emergency department in our hospital. Finally, while the small number of positive CT and MRI findings shows the low diagnostic yield in these neurological symptoms, it also limits the generalizability of our findings.

## Conclusions

Syncope in the ED is generally benign, although a substantial fraction of patients harbors serious neurologic disease. Our data suggest that the identification of predictive variables for the abnormal imaging findings has a potential to decrease the routine use of head CT and MRI in patients presenting to the ED with syncope.

## Abbreviation

CT: Computed tomography
ED: Emergency department
INR: International normalized ratio
MRI: Magnetic resonance imaging
PT: Prothrombin time
PTT: Partial thromboplastin time
